# Correction: Solomon et al. Effects of Platelet-Rich Osteoconductive–Osteoinductive Allograft Compound on Tunnel Widening of ACL Reconstruction: A Randomized Blind Analysis Study. *Pathophysiology* 2022, *29*, 394–404

**DOI:** 10.3390/pathophysiology32030042

**Published:** 2025-08-26

**Authors:** Ruth Solomon, Jan Pieter Hommen, Francesco Travascio

**Affiliations:** 1 Department of Industrial and System Engineering, University of Miami, Coral Gables, FL 33146, USA; r.solomon5@umiami.edu; 2 Department of Orthopedics, Florida International University, Miami, FL 33199, USA; jph@drhommen.com; 3 Department of Mechanical and Aerospace Engineering, University of Miami, Coral Gables, FL 33146, USA; 4 Department of Orthopaedic Surgery, University of Miami, Miami, FL 33136, USA; 5 Max Biedermann Institute for Biomechanics at Mount Sinai Medical Center, Miami Beach, FL 33140, USA

In the original publication [1], there was a mistake in Table 1 as published. Table 1 included 2 subjects in the age range who were excluded due to age. The corrected Table 1 appears below. The authors state that the scientific conclusions are unaffected. This correction was approved by the Academic Editor. The original publication has also been updated.

## Figures and Tables

**Table 1:** Summary of the demographic data of participants.

	OIC (StimuBlast^®^/PRP)	Control
**Male**	8	7
**Female**	5	7
**Age at Time of Surgery**	32.7 ± 10.5	32.7 ± 12.7 y.o.
**Age Range**	18 to 50 y.o.	18 to 55 y.o.
